# Supplementary material for: Discovery of Novel Spike Inhibitors against SARS-CoV-2 Infection
Source: Int J Mol Sci. 2024 Jun 1;25(11):6105. doi: 10.3390/ijms25116105 (PMC11172837; doi:10.3390/ijms25116105)
Supplement: Supplementary file 1 [file ijms-25-06105-s001.zip › ijms-3016052-supplementary.pdf]

# Discovery of Novel Spike Inhibitors against SARS-CoV-2 Infection

**Li-Te Tai [1], Cheng-Yun Yeh [2], Yu-Jen Chang [3], Ju-Fang Liu [4], Kai-Cheng Hsu [5], Ju-Chien Cheng [6,*] and Chih-Hao Lu [7,8,9,*]**

[1] Industrial Development Graduate Program of College of Biological Science and Technology, National Yang Ming Chiao Tung University, Hsinchu 300193, Taiwan; duncan19950626@gmail.com
[2] Graduate Institute of Biomedical Sciences, China Medical University, Taichung 404333, Taiwan; simplefeeling8211@gmail.com
[3] The Ph.D. Program of Biotechnology and Biomedical Industry, China Medical University, Taichung 404333, Taiwan; uzang199431465@gmail.com
[4] School of Oral Hygiene, College of Oral Medicine, Taipei Medical University, Taipei 110301, Taiwan; jufangliu@tmu.edu.tw
[5] Graduate Institute of Cancer Biology and Drug Discovery, Taipei Medical University, Taipei 110301, Taiwan; piki@tmu.edu.tw
[6] Department of Medical Laboratory Science and Biotechnology, China Medical University, Taichung 404333, Taiwan
[7] Institute of Bioinformatics and Systems Biology, National Yang Ming Chiao Tung University, Hsinchu 300193, Taiwan
[8] Department of Biological Science and Technology, National Yang Ming Chiao Tung University, Hsinchu 300193, Taiwan
[9] Center for Intelligent Drug Systems and Smart Bio-Devices (IDS2B), National Yang Ming Chiao Tung University, Hsinchu 300193, Taiwan
* Correspondence: jccheng@mail.cmu.edu.tw (J.-C.C.); chlu@nycu.edu.tw (C.-H.L.)

**Supplementary Table S1.**

Ten NCI compounds were verified after virtual screening, each accompanied by its respective identification number and reference code within the article.

| Compounds | Code |
|---|---|
| NSC103846 | C1 |
| NSC121365 | C2 |
| NSC121969 | C3 |
| NSC237531 | C4 |
| NSC614438 | C5 |
| NSC614440 | C6 |
| NSC648665 | C7 |
| NSC660825 | C8 |
| NSC660826 | C9 |
| NSC660827 | C10 |

**Supplementary Table S2.** Pseudovirus-based inhibiton assay of the ten purchased compounds (C1-C10). The RLU (%) values were calculated using the following equation: RLU (%) = (A / A0) × 100%, where A represents the RLUs of the experimental groups and A0 represents the RLUs of the DMSO group. The data are presented as means ± standard deviation (SD).

| Compounds | RLU(% of control) |
|---|---|
| C1 | 193.0 ± 25.6 |
| C2 | 26.3 ± 3.2 |
| C3 | 96.3 ± 15.0 |
| C4 | 92.3 ± 15.5 |
| C5 | 109.0 ± 11.3 |
| C6 | 57.3 ± 2.9 |
| C7 | 96.3 ± 12.6 |
| C8 | 22.0 ± 2.0 |
| C9 | 41.0 ± 6.2 |
| C10 | 23.3 ± 0.6 |

**Supplementay Table S3.** The relative cell viability percentages for C2, C8, and C10 were determined based on treatments with five concentrations: 100 μM, 200 μM, 300 μM, 400 μM, and 500 μM. Data were normalized to the viability of the control group treated with DMSO. The results are presented as means ± standard deviation (SD).

| Compounds | 100 μM | 200 μM | 300 μM | 400 μM | 500 μM |
|---|---|---|---|---|---|
| C2 | 123.0±9.8 | 117.0±8.2 | 96.7±0.6 | 93.7±1.5 | 91.7±1.2 |
| C8 | 130.7±3.2 | 123.0±2.0 | 105.0±7.0 | 94.7±1.5 | 91.3±1.2 |
| C10 | 130.0±4.0 | 111.3±10.3 | 101.0±1.0 | 91.0±1.0 | 91.7±2.1 |

**Supplementay Table S4.** The inhibition percentages for C2, C8, and C10 were determined based on treatments with five concentrations: 100 μM, 50 μM, 25 μM, 12.5 μM, and 6.25 μM. The results are presented as means ± standard deviation (SD).

| **Compounds** | **100 μM** | **50 μM** | **25 μM** | **12.5 μM** | **6.25 μM** |
|---|---|---|---|---|---|
| C2 | 86.3±1.5 | 78.7±3.2 | 73.7±3.2 | 65.7±3.1 | 28.0±2.6 |
| C8 | 92.7±3.1 | 83.3±4.9 | 78.0±2.0 | 75.0±2.0 | 38.3±0.6 |
| C10 | 93.7±5.9 | 81.3±3.5 | 76.7±0.6 | 72.3±2.1 | 35.7±1.5 |

**Supplementay Table S5.**

The nine analogs from C2 and C8, each accompanied by their respective identification number and reference code within the article.

| Compounds | Code |
|---|---|
| NSC121360 | C2.1 |
| NSC121362 | C2.2 |
| NSC630360 | C2.3 |
| NSC645888 | C2.4 |
| NSC368342 | C8.1 |
| NSC660824 | C8.2 |
| NSC660828 | C8.3 |
| NSC660830 | C8.4 |
| NSC660831 | C8.5 |

**Supplementay Table S6.** Pseudovirus-based inhibiton assay of the C2 and C8 analogs. The RLU (%) values were calculated using the following equation: RLU (%) = (A / A0) × 100%, where A represents the RLUs of the experimental groups and A0 represents the RLUs of the DMSO group. The data are presented as means ± standard deviation (SD).

| **Compounds** | **RLU(% of control)** |
|---|---|
| C2.1 | 33.3 ± 7.8 |
| C2.2 | 51.0 ± 7.0 |
| C8.1 | 118.3 ± 7.6 |
| C8.2 | 2.7 ± 0.6 |
| C8.3 | 15.0 ± 9.5 |
| C8.4 | 15.3 ± 7.2 |
| C8.5 | 29.3 ± 4.6 |

**Supplementay Table S7.** The relative cell viability percentages for C8.2 were determined based on treatments with five concentrations: 100 μM, 200 μM, 300 μM, 400 μM, and 500 μM. Data were normalized to the viability of the control group treated with DMSO. The results are presented as means ± standard deviation (SD).

| **Compounds** | **100 μM** | **200 μM** | **300 μM** | **400 μM** | **500 μM** |
|---|---|---|---|---|---|
| C8.2 | 136.0±11.1 | 132.7±8.0 | 127.7±8.6 | 118.0±7.0 | 106.3±8.5 |

**Supplementay Table S8.** The inhibition percentages for C8.2 were determined based on treatments with five concentrations: 100 µM, 50 µM, 25 µM, 12.5 µM, and 6.25 µM. The results are presented as means ± standard deviation (SD).

| **Compounds** | **100 µM** | **50 µM** | **25 µM** | **12.5 µM** | **6.25 µM** |
|---|---|---|---|---|---|
| C8.2 | 97.3±0.6 | 94.0±1.0 | 93.0±4.6 | 84.0±2.6 | 43.7±1.2 |

**Supplementary Table S9.** The pseudovirus-based inhibiton assay was conducted using the BA.1 strain with C2, C8, C10, and their analogs. The RLU (%) values were calculated using the following equation: RLU (%) = (A / A0) × 100%, where A represents the RLUs of the experimental groups and A0 represents the RLUs of the DMSO group. The data are presented as means ± standard deviation (SD).

| **Compounds** | **RLU(% of control)** |
|---|---|
| C2 | 26.0 ± 3.6 |
| C8 | 26.7 ± 1.5 |
| C10 | 24.7 ± 1.5 |
| C2.1 | 38.3 ± 1.2 |
| C2.2 | 45.0 ± 5.6 |
| C8.1 | 88.3 ± 11.0 |
| C8.2 | 14.0 ± 1.0 |
| C8.3 | 34.0 ± 4.6 |
| C8.4 | 37.3 ± 1.5 |
| C8.5 | 51.3 ± 1.5 |

**Supplementay Table S10.** The inhibition percentages for C8.2 on the BA.1 strain were determined based on treatments with five concentrations: 100 μM, 50 μM, 25 μM, 12.5 μM, and 6.25 μM. The results are presented as means ± standard deviation (SD).

| **Compounds** | **100 μM** | **50 μM** | **25 μM** | **12.5 μM** | **6.25 μM** |
|---|---|---|---|---|---|
| **C8.2** | 97.3±0.3 | 93.9±1.0 | 86.0±1.0 | 77.4±1.7 | 26.2±0.5 |

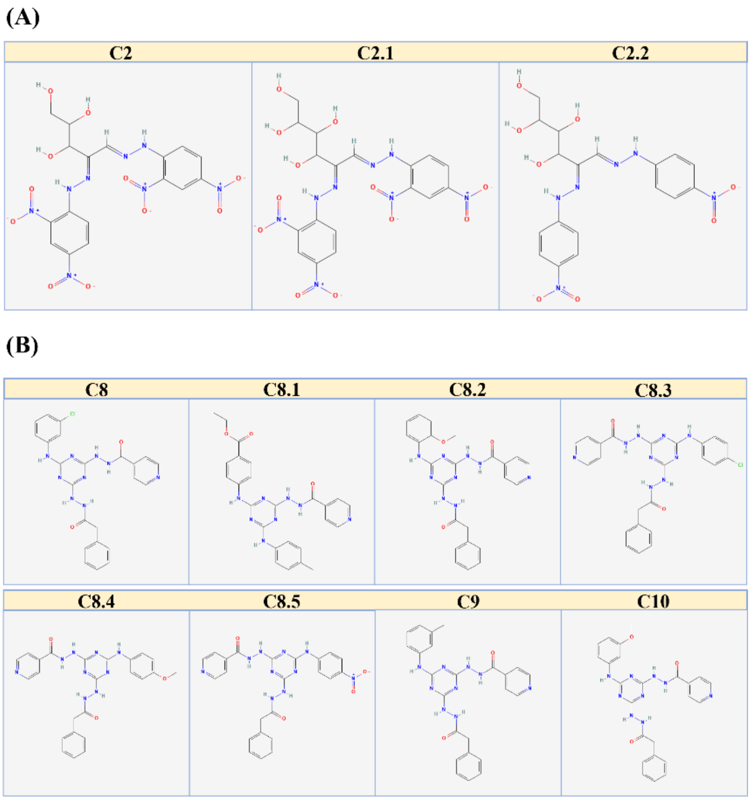


**Supplementary Figure S1.** (A) Structures of compound C2 and the C2 analogs. (B) Structures of compounds C2, C9, C10, and the C8 analogs.
